# Rhetoric, Reality and Racism: The Governance of Aboriginal and Torres Strait Islander Health Workers in a State Government Health Service in Australia

**DOI:** 10.34172/ijhpm.2022.6750

**Published:** 2022-05-09

**Authors:** Stephanie M. Topp, Josslyn Tully, Rachel Cummins, Veronica Graham, Aryati Yashadhana, Lana Elliott, Sean Taylor

**Affiliations:** ^1^College of Public Health Medical and Veterinary Sciences, James Cook University, Townsville, QLD, Australia.; ^2^Torres and Cape Hospital and Health Services (TCHSS), Cairns, QLD, Australia.; ^3^Centre for Primary Health Care & Equity, University of New South Wales, Sydney, NSW, Australia.; ^4^School of Population Health, UNSW, Sydney, NSW, Australia.; ^5^School of Social Sciences, UNSW, Sydney, NSW, Australia.; ^6^School of Public Health and Social Work, Queensland University of Technology (QUT), Brisbane, QLD, Australia.; ^7^NT Health, Darwin, NT, Australia.; ^8^Menzies School of Health Research, Darwin, NT, Australia.

**Keywords:** Indigenous, Governance, Community Health Workers, Australia, Health Workforce

## Abstract

**Background:** In northern Australia, Aboriginal and Torres Strait Islander Health Workers (A&TSIHWs) are unique members of nominally integrated teams of primary care professionals. Spurred by research documenting ongoing structural violence experienced by Indigenous health providers and more recent challenges to recruitment and retention of A&TSIHWs, this study aimed to explore whether the governance of the A&TSIHW role supports full and meaningful participation.

**Methods:** The qualitative study was co-designed by a team of Aboriginal, Torres Strait Islander and non-Indigenous collaborators. Data collection comprised document review and interviews with A&TSIHWs (n=51), clinicians (n=19) community members (n=8) and administrators (n=5) in a north Queensland health district. We analysed governance at multiple levels (regulatory, organisational, and socio-cultural) and used critical race theory to deepen exploration of the role of race and racism in shaping it.

**Results:** Governance of the A&TSIHW role occurs within a health system where racism is built into, and amplified by, formal and informal rules at all levels. Racially discriminatory structures such as the previous but long-standing relegation of A&TSIHW into the same career stream as cleaners were mirrored in discriminatory rules and managerial practices such as an absence of career-specific corporate support and limited opportunities to participate in, or represent to, key leadership groups. These interacted with and helped perpetuate workplace norms permissive of disrespect and abuse by non-Indigenous professionals. Ongoing resistance to the structural violence required of, and demonstrated by A&TSIHWs speaks to the gap between rhetoric and reality of governance for A&TSIHWs.

**Conclusion:** Strengthening governance to support A&TSIHWs requires critical attention be given to the role of race and racism in regulatory structures, organisational practice, and inter-professional relationships. Addressing all domains will be essential to achieve systemic change that recognises, supports and embeds the unique knowledge, skills and functions of the A&TSIHW role.

## Background

**Key Messages**

** Implications for policy makers**

- Aboriginal and Torres Strait Islander Health Workers (A&TSIHWs) are unique members of the Queensland health workforce, with a distinctive set of skills and knowledge crucial to the delivery of culturally safe healthcare.
- The accelerated rate of retirement or passing of older A&TSIHW over the past decade means failure to act in the near term may result in irreversible loss of cultural and corporate wisdom carried by senior A&TSIHWs.
- But a continued gap between the rhetoric and the reality of supportive governance for A&TSIHWs is impacting the wellbeing and ability to retain members of this critical cadre in the public sector workforce.
- Transformation of the formal and informal rules that embed racial hierarchies within the state health system and that enable racist behaviours at all levels is urgently required.
- 2021 changes to the Hospital and Health Service (HHS) Act represent necessary but insufficient reforms.
- But true transformation requires non-Indigenous leaders and professionals at all levels to engage in critical self reflection regarding their role in co-creating a discriminatory work culture, and build their own understanding of, and respect for diverse medical and cultural knowledges.

** Implications for the public**

In northern Australia, Aboriginal and Torres Strait Islander Health Workers (A&TSIHWs) have played a central role in the delivery of primary healthcare for over five decades. However, numbers of A&TSIHWs have been declining in Queensland and elsewhere, so this study tried to understand where the challenges lie, at the policy, organisational, and facility/clinic level. We found racist rules, policies and work culture to be at the centre of weak governance for this group of professionals. At the policy level, A&TSIHWs receive less pay and fewer benefits than their professional counterparts. Within the health service organisations, A&TSIHWs are limited in opportunity to represent, up-skill or progress in their careers, and have little corporate support to elevate their professional needs. At the facility level, many non-Indigenous professionals do not understand or respect A&TSIHWs’ contribution to the function of the health service. The study provides compelling evidence of the need to challenge and transform the racially defined rules and norms that govern A&TSIHWs if they are to be able to deliver on their role as intended.

Community health workers (CHWs) are popularly conceptualised as a ‘bridge’ between community members and the formal health system.^1^ Although highly heterogeneous from country to country, the role is often defined by a focus on at least one of three functions; service extender, cultural broker, or a more transformative role of ‘change agent.’ Globally over the past decade, the role and potential of CHWs has attracted growing attention, particularly as important contributors to effective primary healthcare and the goal of universal health coverage.^2^ Yet for CHW programmes embedded within state health systems many challenges remain. As their bridging function implies, CHWs operate in a space between the formal health system and their communities, a space containing multiple and sometimes conflicting worldviews and priorities.^3^ There is consequently a need for governance strategies that account for roles, relationships and identities not wholly defined by the dominant biomedical knowledge or service models more familiar to doctors, nurses and other health professionals. As Schneider notes, “the interface and relationships of CHWs with the formal [health] system, often described as precarious, needs to be defined and actively managed.”^2^

 In Australia, Aboriginal and Torres Strait Islander Health Workers (A&TSIHWs) have played a central role in the delivery of primary care services in northern jurisdictions for over five decades.^4,5^ A&TSIHWs usually work within primary care clinics as part of an integrated team of professionals responsible for promoting client engagement and self-management.^6^ Distinct from other health professionals, however, the role of A&TSIHW can only be occupied by an Aboriginal and/or Torres Strait Islander person making it the only racialised health profession in the Australian health system. The role spans three major functions including: (*i*) clinical service delivery, (*ii*) health promotion, and, uniquely in the Australian health workforce, (*iii*) cultural brokerage.^7^ This latter function is grounded in A&TSIHWs’ responsibility for helping Indigenous clients to navigate and mediate tensions within health services that have historically normalised racism and assimilation.^7^

 Previous work has traced the historical beginnings of the A&TSIHW role as leprosarium workers in 1950s Northern Territory, to the medical assistants, health promotion officers and cultural brokers of subsequent decades.^5,8,9^ A&TSIHWs themselves have written about their role in healthcare as extending beyond clinical biomedical expertise, in ways that are often deeply political and indicative of a ‘change agent’ role.^10,11^ The political element of the role as understood by its practitioners stems in part from the experiences associated with a long history of racist policies enacted on Aboriginal and Torres Strait Islander peoples (including systematic segregation, restricted cultural practice and use of language and policies that produced the Stolen Generations^12,13^) which engendered widespread distrust of government and government-run health services among Aboriginal and Torres Strait Islander peoples.^14,15^

 Large disparities in access and health outcome indicators between Aboriginal and Torres Strait Islander and non-Indigenous Australians – which continue to be measured but remain effectively unchanged^16^ – speak to these historical and ongoing experiences of structural violence^17^ and their impact on access to healthcare.^14,18^ Race is constructed through the perception of physical (eg, skin colour) and non-physical (eg, accents, behaviour) signifiers^19^ that reinforce historically embedded stereotypes (eg, Franz Fanon’s ‘schemas’^20^); and *racism* is the expression of historical power relationships, ideology, and forms of discrimination (both structural and interpersonal).^21^ Racism is often realised through whiteness, not as a pigmentation *per se*, but as a social construct that helps to materialise, reinforce and naturalise power through the identification with dominant cultural values.^22^ A recurring theme in analyses by Aboriginal and Torres Strait Islander health practitioners-turned-scholars and other (notably critical race) scholars, is how Aboriginal and Torres Strait Islander patients and providers in Australia have had to continually operate within a health system structured for ‘white possession’^23^ and shot through with more and less overt expressions of racism.^24-27^

###  Policy and Health System Context

 Australia’s health system is jointly managed by federal, and state and territory governments. The Australian government develops national health policy, subsidizes primary care and pharmaceuticals directly through the national Medicare and Pharmaceutical Benefits Schemes, and provides funds to state and territory governments for hospital and health services (HHSs).^28^ State and territory governments fund and manage public hospitals and deliver community-based and preventive services, often in conjunction with local governments. In the state of Queensland, health services are delivered by 16 independent, board-governed statutory HHSs who recruit health professionals based on state government defined career structures and associated certified agreements. Nurses and midwives, doctors, and allied health professionals each have a named career stream (eg, ‘Medical Officer’ and ‘Nursing and Midwifery’). However, until late 2019, A&TSIHWs were classified as part of a general ‘Operational’ career stream alongside a variety of (non-health professional) clerical and cleaner roles.

 Although in recent years federal and state government policies started to set targets for increasing the proportion of Aboriginal and Torres Strait Islander health professionals,^29-31^ recruitment and retention of A&TSIHWs, particularly in the north, has been a challenge. Research drawing on Australian Bureau of Statistics data suggests a modest increase in national numbers of centrally-documented A&TSIHWs during the past decade,^32^ but figures indicate a downward trend since a peak in the early 2000s.^33^ According to one recent estimate, numbers of A&TSIHWs were set to decline from 1600 nationally in 2019 to 1300 by 2024.^33^ A 2019 government-commissioned review of the A&TSIHW career structure in the state of Queensland observed that, despite a framework acknowledging the central place of this role to the delivery of culturally safe, accessible and responsive healthcare, *“there is evidence … to suggest that full participation is not being achieved.”*^34^

 The aim of the current study was to investigate from the perspective of those working as A&TSIHWs in the context described above, whether and how the governance of A&TSIHWs in Queensland’s public health system supports full and meaningful participation. We understood full and meaningful participation to mean A&TSIHWs being able to perform all facets of their role as intended, with the support and respect of colleagues, supervisors and the broader system.

## Methods

###  Theoretical Positioning

 The ontological positioning of the project was anchored in the principles of critical realism, which aim to produce transformative change through identifying and explaining the interactions between context, and mechanisms that lead to specific outcomes in specific places.^35^ We then drew on theories of governance, specifically Schneider, which explains how health workforce governance is “*a distributed function, negotiated across levels of the health system and as encompassing analytic, managerial, technical and political roles and capacities*.”^2^ Given the social and political context already described, a key reason for adopting a governance lens was to enable exploration of the way such ‘negotiated and deliberative processes’ across multiple interfaces (eg, between policy-makers, district and facility managers, health workers and communities) give expression or otherwise to longstanding patterns of institutional racism. Schneider among others,^36^ emphasizes that the ‘rules’ – both formal and informal – are ‘*expressed in relationships amongst these actors*.’^2^ We thus understood governance of A&TSIHWs as more than the technical design of professional roles or formal mechanisms of oversight – although these are part of it – but also the vertical and horizontal relationships of A&TSIHW and the ‘strategic and tactical ability’ of actors within those relationships to steer implementation in certain directions. Given the A&TSIHW role is the only identified (racialised) health professional role in the Queensland health workforce, we drew on critical race theory to deepen analysis, by exploring the role of race and racism in the production and reproduction of these formal and informal rules, and their influence on crucial relationships that steer the A&TSIHW role and its provision of culturally safe care.

###  Study Setting

 The northern-most service district in Queensland is the Torres and Cape HHS, responsible for 27 000 people, of whom approximately 64% identify as Aboriginal and/or Torres Strait Islander.^37^ Torres and Cape HHS is responsible for four secondary-level hospitals and 32 primary care centres across 130 000 square kilometers^37^ (an area the size of England). Torres and Cape HHS also has the highest number of A&TSIHWs of any local health district in Australia, with 196 occupied A&TSIHW positions comprising 131 (67%) permanent, 35 (17%) temporary and 30 (15%) casual roles at the time of data collection.^38^ At that time, 38 (19%) of the total A&TSIHW positions and 25 (20%) of the permanent A&TSIHW positions in the region were not occupied.

 The current Torres and Cape HHS comprises two formerly separate regions that were merged in 2014 as part of state-wide push for administrative efficiency. That merge followed an earlier set of radical government-wide austerity measures in 2012, that forced the state health system to cut $326M (equivalent to 4142 jobs) from the budget.^39^ Responsibility for the largest share of budget cuts was passed on to HHSs. In the then separate Cape York HHS, the budget cuts resulted in many entry-level A&TSIHW roles being disestablished. In Torres Strait and Northern Peninsula HHS, a unique service model^40^ based on the principles of primary healthcare where A&TSIHWs positioned as clinic managers or higher had substantial influence, the cuts were viewed by some as an opportunity to ‘clean house’; redundancy packages were targeted at senior A&TSIHWs, whose positions were subsequently disestablished. From a high of 300 A&TSIHW positions in the combined Torres Strait and Cape York regions in 2012, the merged HHS now has just under 200.^38^

###  Study Design

 Consideration for Indigenist research principles including the centring of Aboriginal and Torres Strait Islander peoples’ voices, experiences and interests, and a foregrounding of resistance and political integrity^41^ were at the heart of this project.

 Recognising the marginalisation of A&TSIHW’s voices in both peer reviewed and grey literature to date,^24^ this work was deliberately created and conducted with A&TSIHWs at the centre, including being co-designed and undertaken by a team that included Aboriginal (JT, RC) and Torres Strait Islander (ST) collaborators, all of whom were current or past A&TSIHWs, and four non-Indigenous researchers (SMT, VG, LE, AY).

 The project was a collaboration between researchers from James Cook University and the Aboriginal and Torres Strait Islander Health directorate of the Torres and Cape HHS. Aboriginal and Torres Strait Islander collaborators employed by the HHS were not involved in data collection, but provided intellectual and cultural leadership in study design, as well as advising on history, local context and culturally safe research strategies.

 The study was conducted across four major phases: (*i*) in-person consultation regarding research focus, design and methods with prospective A&TSIHWs participants in all prospective study sites, (*ii*) formal ethical approvals sought followed by in-person interviews and policy review, (*iii*) preliminary investigator analysis and sharing and member checking of interpretation with A&TSIHW study participants, and (*iv*) finalisation and reporting back of findings to all A&TSIHW in the region, and other Torres and Cape HHS and external stakeholders.

###  Sampling

 Interviews were conducted with A&TSIHWs (n = 51), nurses and doctors (n = 19) community members (n = 8) from towns linked to primary health clinics and hospitals in the Torres and Cape HHS catchment; as well as 5 key informants and administrators from the state government.

 Purposive sampling based on pre-study consultation visits was used to recruit A&TSIHWs and non-Indigenous clinical professionals working in the study clinic sites. Community members and all A&TSIHWs in participating sites were consulted during pre-study visits to each site, which informed data collection approaches including formulation of recruitment and dissemination processes. Participants were informed of the interview visit dates in advance and could choose to participate or not. Following guidance on culturally safe research, the recruitment of community members was reliant on direct referral by local A&TSIHW. Recruitment of key informants was purposive based on expertise or experiences and interviews were conducted by one Aboriginal (RC) and two non-Indigenous team members (SMT, VG). RC, JT and ST provided cultural advice and guidance throughout. Interviews explored A&TSIHWs’ motivations for working in the role; and perceptions and experiences of the support provided by formal and informal relationships in the regulatory, organisational and socio-cultural domain. Interviews with non-Indigenous clinicians explored their understanding of the value and purpose of A&TSIHW, and perceptions regarding barriers and enablers to their effective integration and performance in primary healthcare teams. Community member interviews explored their understanding of the value and purpose of the A&TSIHW role and perceptions of its stewardship in the context of current community health needs. Key stakeholder interviews asked similar questions and often included an additional focus on the impact of historical and recent policy and organisational reforms on the A&TSIHW role. All interviews were conducted in English, recorded (with the exception of four interviews where extensive notes were taken), and transcribed verbatim and uploaded into NVivo V.12 (QSR International, 2015).

###  Analysis

 Analysis was conducted with and guided by a concern for centring A&TSIHWs’ voices and the principles of resistance and political integrity.^41^ While the research question queried A&TSIHWs’ ‘lack of full participation’ for example, our analytical focus was honed by the significance A&TSIHWs’ status as the only ‘identified’ (racialised) role operating in predominantly non-Indigenous professional setting; the need to understand how structural and relational violence might be inhibiting their participation; as well as how A&TSIHWs themselves were enacting resistance in the face of such experiences.

 An iterative approach to data analysis emphasised collaboration with A&TSIHWs over three stages including (*i*) initial pre-study consultation and permissions sought from local councils or community groups, (*ii*) careful review of interview transcripts and provision of corrections or additions where study participants so chose and (*iii*) opportunities for feedback and critique of preliminary analyses. Early rounds of analysis were grounded and inductive with themes identified over several rounds, summarised and presented to A&TSIHWs in person or via video conference and interpretations refined based on feedback. Themes were later examined for fit against Aboriginal and Torres Strait Islander^42,43^ and institutional governance^2,36,44,45^ frameworks and with reference to key concepts – namely ‘white possession’ – drawn from critical race scholarship.^23^ Both preliminary and latter analyses were subject to interrogation by individuals active in the A&TSIHW role.

## Results

 Findings are presented according to inductive themes, organised within three levels of governance: the regulatory level encompassing state-wide policies and structures that influence the A&TSIHW career; the organisational level, encompassing managerial rules and norms that influence A&TSIHWs function within the districts (HHSs) who employ them; and the socio-cultural level, which focuses on the rules and norms of facility work culture and relationships. These levels were used as an organising heuristic recognising that, in reality, health system are complex social systems in which each level or domain of governance is tightly linked to all others. Quotes are attributed to individuals by profession-type (A&TSIHW, Nurse, Medical Officer) and a sequential ID.

###  Regulatory

####  Career Stream and Certified Agreements

 At the time of data collection A&TSIHWs were still classified as part of a general ‘Operational’ professional stream alongside clerical and non-health professionals including cleaners. Almost all A&TSIHW participants described this professional classification in a steam of employees many of whom did not require professional or cultural competencies and with far less complex responsibilities as making them feel ‘overlooked,’ ‘sidelined,’ and ‘undervalued.’

 “*We’re operational stream and we’re not cleaners, you know, I disagree with that so badly. I just want to change streams” *[A&TSIHW, #3].

 Negotiation of a new Certified Agreement in 2019 created a temporary ‘Health Worker’ career stream and was noted as belated but important progress. Yet even under this new agreement, AT&SIHWs lack access to a wide range of benefits ns standard for other professionals, including remote housing and more generous professional development. Table compares the guaranteed benefits linked to A&TSIHW roles (under the “new” 2019 certified agreement) compared to nurses and midwives, and medical officers. Of particular concern to study participants was the disparity in provision of Housing Benefits for remote area service. In remote Aboriginal and Torres Strait Islander communities in Queensland, housing is government owned (used for service personnel) or community-controlled. This means that there may be no rental properties, or, where rental properties are available they are expensive (eg, 3 bedroom homes average AUD-650 per week). Certified Agreements for all other health professionals make provision for government housing (or rental subsidy) as a standard part of any remote area contract. For A&TSIHWs however, such entitlements are at the discretion of the employing HHS and rental subsidy provisions substantially less.

**Table:** Comparison of Award Conditions for Different Professionals Employed by Queensland Health

**Award**	**Aboriginal and/or Torres Strait Islander Health Worker**^a^	**RN, RM and CNC**^b^	**RMOs and SMOs**^c^
Base salary	Range: Health worker stream level 1.1 from 01/10/2019: $40 000 p/a. Operational stream level 9.3 from 01/10/2018: $114 000 p/a.	RN/RM Range: Level 5.1 from 01/04/2018: $65 899 p/a. Level 5.7 from 01/04/2018: $88 474 p/a. CNC Range: Level 7.1 from 01/04/2018: $110 499 p/a. Level 7.4 from 01/09/2018: $119 964 p/a.	Range: Level 1 from 01/07/2018: $75 137 p/a. Level 29 from 01/09/2018: $244 198 p/a.
Overtime and TOIL	Not eligible for overtime. TOIL up to a maximum of 10 days in any one year. TOIL entitlements lapse if not used within 6 months.	Overtime provisions: ♦ Monday-Friday: 1.5x first 3 h; 2x thereafter. ♦ Saturday: 1.5 first 3 h; 2x thereafter (min. 2 h). ♦ Sunday: 2x time (min. 2 h). ♦ Employees accrued day off: 1.5 first 3 h; 2x thereafter (min. 2 h). Provisions for TOIL per A&TSIHW.	RMOs a­­­nd SMOs access the same overtime provisions as RN, RM and CNC employees.
Remote accommodation	May access QH accommodation if eligible, *subject to permissions *from health service executive. Where accommodation not provided, “Special Allowance” $38.66/week.	Up to 17 months rent-free QH accommodation. Or weekly allowance of $82.50 pw for up to 17 months if QH accommodation­­­ is unavailable. Health service chief executive (or delegate) has discretion to extend accommodation assistance.	RMOs a­­­nd SMOs access the same accommodation assistance provisions as RN, RM and CNC employees.
Rural and remote allowances	Not eligible for this allowance.	Remote area nursing incentive package benefits per categories below, plus all relocation expenses. If providing short-term relief to rural & remote facilities, accommodation plus meals (or allowances) provided for up to 4 weeks.	RMOs and SMOs entitled to “Inaccessibility Allowance” of between $34 500-$48 300 p/a. SMOs entitled to “Regional and Rural Attraction Allowance” increasing base salary 5%-15% depending on remoteness.
Travel benefits	Eligible for TOIL of travel.	Employees (and spouse and dependents) living in designated remote areas get 2x return airfares p/a. Cash equivalent (if not travelling by air) and mileage allowances (if located remotely) are available.	There are no specific provisions but the “Inaccessibility Allowance” (see above) under the Agreement intended to support travel for recreation in addition to other personal and family costs.
PD	Permanent, or temporary with more than 12 months continuous service A&TSIHW and A&TSIHP are entitled to interim PD allowance, pro rata, based on qualification and categories of HHS facilities. Allowance ranges from $1043 - $2320 p/a. Eligible staff granted paid leave, pro rata for part time, of 2 days p/a (level 005 or above, or A&TSIHP) or 3 days p/a. (A&TSIHW up to Lv. 004) for approved PD activities.	PD allowance of b/w $1809-$3016 p/a (From 2016). Employees working in designated remote areas entitled to a minimum of 2 weeks PD leave p/a plus travel as required and enrolment and conference costs for approved courses and conferences.	RMOs are entitled to 1.6 weeks PD leave p/a. plus additional 0.6 weeks p/a. for remote and rural posting, accumulated for up to 5 years. PD allowance of $222 p/a (From 2018). RMOs in vocational training program entitled to “Vocational Training Subsidy” $3670 p/a., paid fortnightly. “Examination leave” separate from other entitlements. SMOs SMOs accrue 3.6 weeks of PD Leave p/a. for max. 10 years. Annual PD allowance, paid fortnightly, of $20 500.
Bonuses	- From September 1, 2019 an Aboriginal and Torres Strait Islander Health Workers special allowance paid fortnightly: $89.42, with 2.5% p/a increase.	Annual isolation allowance based on length remote service: After 1 year: $3500. After 2 years: $10 500. Each year after 3 years: $7000.	Nil provisions.
Other substantive benefits	Nil provisions.	On-call loadings as follows: $46.97 per on-call period (weekends, public holidays days off). $25.67 per on-call period (Monday-Friday).	On call allowance. “Clinical Support Time”: protected time during ordinary hours for duties not directly related to patient care (incl. teaching, research, clinical governance, admin and other).

Abbreviations: P/A, per annum; QH, Queensland Health; b/w, between; RN, registered nurse; RM, registered midwife; CNC, clinical nurse consultant; RMOs, resident medical officers; SMOs, senior medical officers; TOIL, time off in lieu; A&TSIHW, Aboriginal and Torres Strait Islander Health Worker; PD, Professional development; A&TSIHP, Aboriginal and Torres Strait Islander Health Practitioner. ^a^ Aboriginal and Torres Strait Islander Health Workforce (Queensland Health) Certified Agreement (No. 1) 2019. ^b^ Nurses and Midwives (QH and Department of Education) Certified Agreement (EB10) 2018. ^c^ Medical Officers’ (QH) Certified Agreement (No.5) 2018.

 The exclusion of housing benefits from A&TSIHW contracts is based on the assumption that A&TSIHWs only work in their community of birth, and relatedly, that they have automatic access to community-controlled accommodation. However, community-controlled housing is limited and overcrowded. Several participants were currently, or had ­­previously been, on a waiting list for housing for several years, living in crowded family houses or ‘couch surfing’ while carrying out their professional role. These circumstances made it difficult to rest, recuperate or – where necessary – study. Many described their frustration with the loss of dignity and productivity that accompanied these circumstances.

 “*So originally, when I first came up here, I was staying with family members, which is a stress. But then I was staying – renting accommodation at the Aged Care Hostel, which is also a stress” *[A&TSIHW, #6].

*“You’re stuck on the island and […] you had to stay with your parents and there’s brothers and sisters and you’re trying to organise yourself with the centre and the – you don’t ever have rest” *[A&TSIHW, #60].

 “*That young fella [A&TSIHW trainee], he lived in two different houses. One was [a party house]. How is he going to sleep at night when everyone’s partying all night? […] We tried to find somewhere else for him. Queensland Health wouldn’t pay for anywhere” *[A&TSIHW, #4].

 Both A&TSIHWs and non-Indigenous participants reported lack of housing as impeding regional workforce mobility among A&TSIHWs, a critical feature of both individual and region-wide capacity development. Ability to recruit senior and experienced A&TSIHWs who could provide essential mentorship for junior recruits was an essential feature of maintaining the workforce.

 “*I know the old rationale for that is, we like to recruit [A*&*TSIHWs] out of the community. You probably heard it a hundred times. And that’s all good, if it works. But what if you can’t get the Health Workers out of the communities? […] You need the experienced Health Workers to nurture the early ones, the learning ones. And you want good experienced Health Workers to do that” *[Medical Officer, #52].

 Mobility was also described by A&TSIHWs as important for their own technical and professional skills, which evolved through experience of working in different teams and settings. Shifting location was additionally noted as one of the few mechanisms available to help A&TSIHWs ‘decompress’ from the mental health impacts of serving their close relatives and friends, which accumulated when based in their own communities.

 Several participants described having not applied to, or turned down offers of a role in communities where they would have had to rely on extended family members or friends for a bed. Lack of housing was described as damaging recruitment and retention of A&TSIHW, and inhibiting the best mix of hyper-local and more regionally-experience staff.

 “*We had advertised a lot of positions. But again, a lot of people do apply, but when they ask us is there accommodation attached to it and we said: “no” they refuse. Because we haven’t been filing the positions, then what happens? They [the roles] get taken away” *[A&TSIHW, #47].

####  Qualifications and Career Progression

 Debates and decisions regarding the A&TSIHWs’ minimum qualifications and professional development have a complicated and ongoing history in both state- and federal jurisdictions in Australia.^46-49^ Of relevance to participants at the time of study was the 2012 introduction of a nationally recognised role Aboriginal and Torres Strait Islander Health* Practitioner* (A&TSIHP) with formal registration through the Australia Health Practitioner Regulation Agency (Ahpra). The key distinction between the A&TSIHW and A&TSIHP role which enables ‘registration’ lies with the degree of responsibility for medications handling, linked to a different minimum qualification.^50^

 When introduced in 2012, Ahpra registration for A&TISIHW/P was seen as a first step to harmonising qualifications and expectations across Australia’s various state and territory jurisdictions with potential for improving A&TSIHW mobility. In 2012, some Australian states and territories already had ‘practitioner’ roles in their career structure. Others, including Queensland, however did not, and for 3 years from July 1, 2012 A&TSIHWs employed in Queensland could apply to have their qualifications and experience considered and counted as adequate for registration as a A&TSIHP in a ‘grandfathering’ arrangement.^51^ Many study participants had taken advantage of this arrangement, registering as A&TSIHP with Ahpra, anticipating the introduction of that role in Queensland; additionally, Torres and Cape HHS and the regional Primary Healthcare Network allocated funds to support A&TSIHWs who wanted to complete the new minimum qualification.^50^ Yet, nearly a decade on in 2021, the Queensland Health Career Structure that defines A&TSIHW employment, had not been updated to include the new A&TSIHP role. Disappointment regarding postponement of these oft-promised reforms, and disillusionment at having spent time, energy and money on qualifications and annual Ahpra registration fees, without resultant opportunity to utilise those skills was evident in many participants’ responses.

 “*She’s […] done her [minimum qualification], she’ll be a health practitioner technically. But she’s got to wait for a job to come up, because there’s no jobs here offered in [this clinic] at this point in time [at that level]. Just because you’ve gone and done the study, doesn’t mean they’re going to give you a pay rise. It means you have to sit in a position and wait” *[A&TSIHW, #57].

###  Organisational

####  Corporate Support and Participatory Governance 

 Shifts in the administrative boundaries of HHSs, changes to governance structures that removed middle-managers and budget cuts throughout the 2010s all impacted on recognition and support of A&TSIHWs. Successive budget cuts in 2012 and 2014, for example, were linked to a growing number of “ghost positions,” meaning A&TSIHW positions that existed on paper but were not funded. As one nurse manager noted.

 “*We lost a huge amount of [A*&*TSIHW] positions with the Newman government, and that was detrimental, and certainly I’ve seen some “sly disestablishment” of A*&*TSIHW roles. Yeah, we’ve lost two roles here without the supporting paperwork. We had people in the roles but the funding is gone and there was no - I looked for the paper trail for that and then that seemed - one didn’t exist*” [Nurse, #18].

 Executive level representation of, or for, A&TSIHWs was also weak. At the time of study, the only professional lead for A&TSIHWs was a manager-level position, and formally only responsible for the southern half of the region with no equivalent role in the north. This middle manager position was effectively responsible for supporting all 196 A&TSIHWs across a 130 000 square kilometre region. Key decisions about how many, and what type of A&TSIHW positions were funded, and what type of support they received were frequently made by committees at clinic and district service level that, absent any ATSIHW representation, were divorced from the realities of the profession and the needs of frontline services.

 “*There’s a massive disconnect there […] I feel like finance runs this HHS and it’s not the other way around, so I think there’s a massive imbalance” *[Nurse, #18].

 Lack of representation, support and advocacy within the HHS management structures was described as contributing to a sense of lack of recognition among many A&TSIHWs. Shrinking opportunities for career advancement were also noted and linked to the thin professional support within the HHS.

 “*There’s not enough Indigenous health workers out there that’s being put back into the system with the health. I can’t speak for how upper management deals with this sort of stuff, but I feel that we’re not being heard. […] Our voices have really not been heard across in the health” *[A&TSIHW, #20].

 In 2017 a Principal Advisor Aboriginal and Torres Strait Health position was created within the Torres and Cape HHS, which became the professional lead and formed part of the HHS executive structure. In 2018/2019 the ‘Principal Advisor’ role was further upgraded to the Executive Director Aboriginal and Torres Strait Islander Health. At the time of writing, however, this Executive Director role was vacant and professional support for A&TSIHWs resided again with the Manager of Health Worker services.

###  Socio-Cultural 

####  Performance Culture, Management and Inter-professional Dynamics

 The dominant work culture of the primary health centres was noted by participants to align with biomedical service models and key performance indicators that sidelined the complex work of A&TSIHWs at the cultural interface of health service and community.

 “*Yeah, that’s not recognised, what you do on a day-to-day basis. Like, people are expecting numbers. People are expecting reports handed in. You can’t hand in something where - how do you put it into words? It’s the programs or the databases that they have doesn’t reflect your real work” *[A&TSIHW, #40].

 Various participants highlighted the way government performance indicators recorded ‘instances of care,’ but lacked attention to (and effectively devalued) the timing and quality of interaction. Many described the difficulty of convincing colleagues and managers that time spent with clients, particularly in non-clinic settings was critical to delivering culturally safe and good quality care.

 “*Sometimes, […] you see one client. But that one client, the issues that they have, it doesn’t take 15 minutes. It’s not a doctor’s consult. They’re in here - sometimes they come in the morning, and they leave and it’s like: ‘Oh, sorry I was here all day.’ And that doesn’t reflect on the computer. It doesn’t show. Like, you can’t feed emotions into like the computer and saying like, ‘I had to take the whole day for this client.’ That’s one thing that’s not recognised. It’s all about numbers but it’s not about the quality of the consult. It’s more quantity. And you can’t put that down in the computer” *[A&TSIHW, #41].

 Although A&TSIHWs described their expectations for their role as one of partnership, working as equals alongside other professionals within (more or less) integrated teams, few reported being treated as equal members on a day to day basis. To the contrary, a majority of A&TSIHW participants detailed near-daily experiences of disrespect, micro-management and both explicit and implicit segregation.

 “*I would love us to come as one, instead of us being segregated. We are in a partnership but there’s no partnership. There’s no leadership. You hear upper management making decisions, but when you come back to us, it’s like the decision still hasn’t been made on the ground. We work here. We stayed here. You don’t. So why aren’t you listening? So that’s – we’re not just being – we’re not being heard” *[A&TSIHW, #20].

 Still prevalent confusion among non-Indigenous providers about the nature, purpose and scope of the A&TSIHW role, contributed such attitudes, as evidenced in a range of non-Indigenous professionals’ descriptions:

 “*The role descriptions are quite ambiguous. And I mean you can say that too about a nursing role. But nursing roles have been around for centuries […] So the problem with health workers is: where’s the governance?” *[Nurse, #11].

 “*I look forward to the health workers having a more defined career structure and more regulated. And it’s good for them, and it’s good for everyone to understand the role and know what the role can do” *[Medical Officer, #18].

 “*I think the thing to really know is the role is different everywhere […] it is not a recognised profession or recognised as a whole because the roles are so different” *[A&TSIHW, #13].

 But an underlying tension between nurses and doctors whose professional roles were defined in terms of their health function and A&TSIHWs whose role required the bridging of biomedical and Indigenous world views and knowledge systems was also a factor. Described as task-oriented, clinic-based, and steeped in a biomedical hierarchy, nursing culture in particular was identified by A&TSIHWs and even some nurse-qualified participants as clashing with the Indigenous Ways of Being, Knowing and Doing that shaped A&TSIHWs’ practice.

 “*I think that nursing has a tendency to take over services, that we - yeah, we do, we just - I think that we disempower [A&TSIHWs] in a lot of ways and you certainly see that in some places […] they’ll come in and kind of take over and do everything” *[Nurse, #18]

 “*That’s the thing with RNs when they come out, they don’t listen. As if: ‘oh I’m the boss here and I do this because I’ve got a higher degree.’ And that’s what I told him. I said: “But culturally you don’t. Culturally I have a higher degree” *[A&TSIHW, #38].

 Many A&TSIHWs described the fatigue they experienced when they had to re-orient, reassure and learn how to work with incoming non-Indigenous clinical health professionals on a regular basis.

 “*We’ve been here for how long? We’re part of the furniture sort of stuff, but then A&TSIHWs remains a stable work force within each remote clinic and community. Whereas nurses come, nurses go. Doctors come, doctors go. You know, and then at the end of the day the community suffers because it takes a long time to build up that rapport with community and then they go and leave, you know what I mean? It’s just a vicious circle for that” *[A&TSIHW, #4].

 The knowledge, attitudes and capacity of clinic managers were described as a key factor in the management of inter-professional tensions. Fewer concerns regarding inter-professional conflict were raised among participants in the Torres Strait and Northern Peninsula Area where a home grown model of primary healthcare enabled A&TSIHWs to be appointed to clinic manager roles. In health facilities further south, by contrast, most facility managers were nurses. While several A&TSIHWs described productive and strong relationships with these managers, many more described experiences of bullying and exclusion. Examples included the regulation and monitoring of sign in sheets, including for tea and cigarette breaks as a time management mechanism; being told to curtail community outreach activities that were not ‘core’ to the funded role; and clinic-wide decisions about service delivery or clinic structure that were not communicated to A&TSIHW team members, resulting in misunderstandings with clients and other community members and loss of face.

####  Identity, Trust and Obligations in the Community

 Understanding how to balance cultural protocols with health-professional responsibilities was described as a central but challenging part of the A&TSIWH role. It was perceived as something that developed with age, experience and mentorship, rather than being innate or the product of pre-service qualifications. Critical skills included understanding local cultural protocol; managing cultural and social sensitivities around family contact; knowledge of cultural protocol regarding who could and could not see each other and under what circumstances; and how to manage community members’ feelings of shame and concerns regarding confidentiality for different types of health issues. As one participant noted:

 “*It’s not something you pick up in a textbook and read” *[A&TSIHW, #45].

 Without exception, A&TSIHW participants described mentorship by seniors as critical to their development and success. For those working in their own community, personal and professional challenges arose in relation to the high level of community embeddedness and the impossibility of disengaging from community or family politics. For many, complicated caring responsibilities for immediate and extended family was an ever-present reality. And despite limitations to their formal scope of practice, community members often held wide-ranging expectations of A&TSIHWs, from on-call transport to provision of clinical care.Mentorship around how to manage those expectations and still achieve the profession’s goals was essential, as one participant observed:

 “*I will say the old ones, you can’t beat the old ones. They just knew how to ‘get there’ with the people” *[A&TSIHW, #60].

 For those who were working outside their community, however, different challenge arose, including the need to build rapport and trust with local clients. While most participants agreed that this could be done, and that being Aboriginal and/or Torres Strait Islander was an important prerequisite, it still took time and investment to understand the nuances of the local culture. In such circumstances access to mentoring was particularly important.

 “*Ever since I’ve been to the community I’ve had good support, yeah. But, slowly, slowly. It’s not like when I first started, there was nothing. I know these people, but they didn’t engage with me, sort of thing, so it took me, like, nearly one and a half years, three years till now” *[A&TSIHW, #50].

 “*I’m talking about this old healer […] He introduced the culture to me, and every time I needed some cultural advice I’d give him a call and I’d tell him, ‘Listen, this old fella needs to talk about this and that, how do I go about it?’ […] So he was like my cultural mentor here” *[A&TSIHW, #2].

 Yet mentorship by senior A&TSIHWs on how to manage this ‘interface’ between health service and community expectations was difficult to secure. Participants described how as the numbers of A&TSIHWs declined, and older A&TSIHWs retired, the opportunities for new and still-developing recruits to be mentored into these important skills were increasingly limited. In situations where a senior A&TSIHW was not present, finding a cultural mentor was up to the individual, with no formal arrangements in place.

####  Stress and Burnout

 Lack of access to mentorship combined with weak professional recognition, workplace bullying, and inability to engage in the work they saw as most important were all factors contributing to the cumulative stress evident in participants’ accounts.

 “*They’re never off work […] they’re always under the eyes of the community. So they go under a lot of stress. The nurses, they can come out, fly out, and come for professional development, stuff like that. But the [A*&*TSIHWs], they can’t […]” *[Nurse, #39].

 “*Oh God how am I going to fit this into words? […] The[y] can say they know what we’re doing, but they’re not here to see […] the frustration and the stress and the depression” *[A&TSIHW, #38].

 Participants also noted the absence of any support for A&TSIHWs to manage and mitigate (anticipatable) psycho-social stressors linked to a role which requires them to continuously bridge community, cultural and professional domains.

 “*They don’t get no sort of support there. No relief workers or anything […]. I think some actually have left because they’re just too tired and they got eventually then burnt out. Nobody help them” *[A&TSIHW, #58].

 For many participants this stress was factor in difficulties retaining individuals in the A&TSIHW role.

 “*People get tired. They get tired, after tiredness comes frustration, after frustration comes anger, and then [they say]: ‘I’m going mate’” *[A&TSIHW, #57].

## Discussion

 “*It is not enough to do the organisational minimum in meeting legal obligations on racial equality, a maximalist approach requires strong leadership and a commitment to institutional transformation” *(p.xiv).^53^

 This study set out to answer whether and how governance of A&TSIHWs supports their participation in the Queensland health workforce.^17,34^ Drawing on the accounts of 82 health professionals including 51 current or former A&TSIHWs in the northern most health district in Queensland, the study explored the governance of A&TSIHW role at the regulatory, organisation and socio-cultural levels of the health system. Across all levels findings point to a continued gap between the rhetoric and reality of governance for A&TSIHWs, noted by Indigenous researchers at least three decades ago,^5^ with race and racism at its core. At the regulatory level, racism is apparent in the inequitable remuneration and incentives that position A&TSIHWs as ‘lesser’ members of the workforce than their nursing, medical or allied health counterparts. Limited leadership for, or of A&TSIHW, at the HHS and facility-level underpin a lack of meaningful workforce integration or professional development that leaves A&TSIHWs sidelined from planning and implementation of key tasks. Overwhelmingly, A&TSIHWs in this study reported experiencing a lack of recognition, emblematic of system of governance that privileges and protects non-Indigenous providers and their worldview.

 An understanding of governance as a distributed function, negotiated across levels of the health system helps to explore the way formal and informal rules in different domains of the health system still constrain A&TSIHWs’ opportunity and autonomy to carry out the role as intended. Bringing a critical race lens to bear further deepens understanding of the way mechanisms of governance are shaped by the ‘white possession’ characteristic of the health system. Findings showed the reality of governance for A&TSIHWs in this setting as one in which racism is built into, and amplified by, both the formal and informal rules. Racially discriminatory structures such as the (previous but long-standing) relegation of A&TSIHW to the operational career stream are reflected in rules and managerial practices at the HHS-level where there remain limited opportunities to represent or advocate for the needs of the role. These structural and organisational forms of exclusion interact with and help perpetuate workplace norms dismissive of non-biomedical knowledge and expertise, and are implicitly permissive of disrespect and abuse of A&TSIHWs by non-Indigenous professionals.^54^ While not always explicitly described as such, the consistency and repeated nature of A&TSIHWs’ experiences of disrespect and exclusion, speak to long-standing racist norms documented since the 1990s, although certainly occurring far earlier.^5,55-58^

 While theories of good governance stipulate the importance of clear leadership and management, and mechanisms that enable participation and feedback,^2^ critical race scholarship draws attention to the way race can structure a system such that ‘owners’ utilise leadership and management structures to protect certain groups while systematically excluding others. Moreton-Robinsone observes of the “*pervasiveness of [Australia’s] white possessiveness [that it] functions through social institutions such as the workplace, operating in everyday intersubjective relationships between Indigenous and white subjects [such that] [t]he right to be here and the sense of belonging it creates are reinforced institutionally, and socially” *(p. 82).^23^ Such ownership was illustrated in the declarations of *non*-Indigenous providers’ in this study who, while acknowledging limited understanding of the full purpose or scope of the A&TSIHW role, were critical of what they felt was its ill-defined nature, and a need for stronger clinical governance and performance accountability. Entitled by a strong sense of belonging within a system that protected their professional and cultural identity, few of these non-Indigenous participants reflected on the tension between their lack of understanding of the role, and their quick prescriptions for how to improve it. Reports of the limited participation of A&TSIHWs that, in part, catalysed this study are thus not a product of value-neutral policies and processes, but rather a system structured by race to produce “*a powerful form of social stratification and means of differentially allocating power, resources and opportunities – advantaging and privileging those considered superior and disadvantaging and excluding those considered inferior*.”^59,60^

 Previous studies have highlighted that the A&TSIHW role is highly beneficial to supporting healthcare accessibility for Aboriginal and Torres Strait Islander clients.^61,62^ But rapid turnover and burn out in this cadre^4,63,64^ inhibit their ability to provide such support and are costly to both the health system and the communities they serve.^64^ Our findings re-emphasise the importance of policy and planning actions (such as those outlined in the Cultural Safety Framework: National Aboriginal and Torres Strait Islander Health Workers and Practitioners Association^65^ and the Indigenous Allied Health Association Cultural Responsiveness Framework^66^) that commit health system leaders to genuine cultural responsivity through governance strategies that actively *dis-embed* ‘white ownership’ and the resultant racism that marks its function. Yet such true transformation is both difficult and rare.

 The late 2021 publication of a Queensland Health Equity Framework^67^ and associated revision of Hospital and Health Boards Act^68^ that aims to ‘actively eliminate racial discrimination and institutional racism’ within the Queensland health system, speaks to some recognition of the imperative to reform. It lists actions including identifying and addressing racist policies and regulations; introducing a zero-tolerance workplace culture to address racism; and increased representation of Aboriginal and Torres Strait Islander peoples in leadership at all workforce levels. However the broader context in which these efforts occur remains profoundly structured by the power of white possession; and true transformation of the type that delegitimises both explicit *and* implicit racial hierarchies requires changing the informal as well as formal rules. This will require significant hard work on the part of non-Indigenous leaders and health professionals who benefit, whether knowingly or not, from the present system. Acknowledgement of their role in co-creating the current work culture; willingness to build knowledge and respect of diverse medical and cultural knowledges^7,69,70^ and critical self-reflection well beyond what individually performed one-off cultural awareness courses can provide are all needed.^58,71-73^ Meanwhile, ongoing resistance to the structural violence required of, and demonstrated by A&TSIHWs in this study, and others,^5,74,75^ signals the scale of the challenge likely to be faced to enact such transformation.

 Findings from this study relate to A&TSIHWs and other professionals working in a sole HHS in the state of Queensland and the particularities of that setting mean their relevance elsewhere should be interpreted with caution. A further limitation was the small number of Aboriginal and Torres Strait Islander clients or community members able to be interviewed, whose perspectives may have enhanced understanding and interpretation of the socio-cultural domain; this was in part due to careful application of ethical procedures relating to the recruitment of non-health professionals into the study. Nonetheless we note how the current research builds on and aligns with previous work illustrative of structural and organisational inequities and systemic racism found in primary care services around Queensland and Australia.^25,44,62,76,77^

## Conclusion

 A large gap exists between the rhetoric and reality of governance for A&TSIHWs in the Queensland health workforce. Critical attention to the role of race and racism in regulatory structures, organisational practice and inter-professional relationships is required to strengthen governance to support A&TSIHWs and to address the deeply rooted barriers that currently prevent full realisation of the role.

## Acknowledgements

 We thank all study participants but particularly the 51 A&TSIHWs who helped shape this research from inception and without whose contributions and trust this work would not have happened. We thank Venessa Curnow for her support throughout including helping share findings widely within and outside Queensland Health. Alex Edelman provided assistance to this study through the production of several data synthesis reports. SMT would like to acknowledge Marta Schaaf and other colleagues present at the ‘Think-in’ on Community Health Worker Voice, Power and Citizens’ Right to Health workshop (June 2017) co-hosted by the Averting Maternal Death and Disability Program at the Mailman School of Public Health, Columbia University and the Accountability Research Centre at American University, as a catalyst for conversations that led to this study.

## Ethical issues

 This study received ethical approval from the Cairns and Hinterland Human Research Ethics Committee (HREC/2018/QCH/45310 – 1290) and James Cook University’s HREC (H7687).

## Competing interests

 Authors declare that they have no competing interests. JT is employed by Torres and Cape Hospital and Health Service. ST was formerly employed by Torres and Cape Hospital and Health Service. The service played no role in the design, analysis or write up of this work.

## Authors’ contributions

 Designed the study: SMT, ST. Provided cultural oversight: ST, JT, and RC. Conducted interviews: SMT, RC, VG. Participated in analysis: SMT, ST, RC, VG, AY, and LE. Wrote first draft of the manuscript; SMT. Provided critical revisions: ST, RC, VG, AY, LE, and RC.

## Funding

 This research was supported by a Career Development Fellowship (SMT) funded by the National Health and Medical Research Council and Hot North Collaboration, GNT1131932; and in-kind support from the Torres and Cape HHS. SMT holds a NHMRC Investigator Award (2020-24) GNT1173004.
